# Validation of IMPROD biparametric MRI in men with clinically suspected prostate cancer: A prospective multi-institutional trial

**DOI:** 10.1371/journal.pmed.1002813

**Published:** 2019-06-03

**Authors:** Ivan Jambor, Janne Verho, Otto Ettala, Juha Knaapila, Pekka Taimen, Kari T. Syvänen, Aida Kiviniemi, Esa Kähkönen, Ileana Montoya Perez, Marjo Seppänen, Antti Rannikko, Outi Oksanen, Jarno Riikonen, Sanna Mari Vimpeli, Tommi Kauko, Harri Merisaari, Markku Kallajoki, Tuomas Mirtti, Tarja Lamminen, Jani Saunavaara, Hannu J. Aronen, Peter J. Boström

**Affiliations:** 1 Department of Radiology, University of Turku, Turku, Finland; 2 Department of Radiology, Icahn School of Medicine at Mount Sinai, New York, New York, United States of America; 3 Medical Imaging Centre of Southwest Finland, Turku University Hospital, Turku, Finland; 4 Department of Urology, University of Turku and Turku University Hospital, Turku, Finland; 5 Institute of Biomedicine, University of Turku, Turku, Finland; 6 Department of Pathology, Turku University Hospital, Turku, Finland; 7 Department of Future Technologies, University of Turku, Turku, Finland; 8 Department of Surgery, Satakunta Central Hospital, Pori, Finland; 9 Department of Urology, Helsinki University and Helsinki University Hospital, Helsinki, Finland; 10 Department of Radiology, Helsinki University Hospital, Helsinki, Finland; 11 Department of Urology, Tampere University Hospital and University of Tampere, Tampere, Finland; 12 Department of Radiology, Tampere University Hospital, Tampere, Finland; 13 Department of Biostatistics, University of Turku, Turku, Finland; 14 Department of Pathology, University of Helsinki, Helsinki, Finland; Peter MacCallum Cancer Centre, AUSTRALIA

## Abstract

**Background:**

Magnetic resonance imaging (MRI) combined with targeted biopsy (TB) is increasingly used in men with clinically suspected prostate cancer (PCa), but the long acquisition times, high costs, and inter-center/reader variability of routine multiparametric prostate MRI limit its wider adoption.

**Methods and findings:**

The aim was to validate a previously developed unique MRI acquisition and reporting protocol, IMPROD biparametric MRI (bpMRI) (NCT01864135), in men with a clinical suspicion of PCa in a multi-institutional trial (NCT02241122). IMPROD bpMRI has average acquisition time of 15 minutes (no endorectal coil, no intravenous contrast use) and consists of T2-weighted imaging and 3 separate diffusion-weighed imaging acquisitions. Between February 1, 2015, and March 31, 2017, 364 men with a clinical suspicion of PCa were enrolled at 4 institutions in Finland. Men with an equivocal to high suspicion (IMPROD bpMRI Likert score 3–5) of PCa had 2 TBs of up to 2 lesions followed by a systematic biopsy (SB). Men with a low to very low suspicion (IMPROD bpMRI Likert score 1–2) had only SB. All data and protocols are freely available. The primary outcome of the trial was diagnostic accuracy—including overall accuracy, sensitivity, specificity, negative predictive value (NPV), and positive predictive value—of IMPROD bpMRI for clinically significant PCa (SPCa), which was defined as a Gleason score ≥ 3 + 4 (Gleason grade group 2 or higher). In total, 338 (338/364, 93%) prospectively enrolled men completed the trial. The accuracy and NPV of IMPROD bpMRI for SPCa were 70% (113/161) and 95% (71/75) (95% CI 87%–98%), respectively. Restricting the biopsy to men with equivocal to highly suspicious IMPROD bpMRI findings would have resulted in a 22% (75/338) reduction in the number of men undergoing biopsy while missing 4 (3%, 4/146) men with SPCa. The main limitation is uncertainty about the true PCa prevalence in the study cohort, since some of the men may have PCa despite having negative biopsy findings.

**Conclusions:**

IMPROD bpMRI demonstrated a high NPV for SPCa in men with a clinical suspicion of PCa in this prospective multi-institutional clinical trial.

**Trial registration:**

ClinicalTrials.gov NCT02241122.

## Abstract 2

Ivan Jambor and colleagues validate a magnetic resonance and reporting program for detecting negative predictive value, reducing the need for biopsies in some men with suspected prostate cancer.

## Author summary

**Why was this study done?:**

- Prostate magnetic resonance imaging (MRI) is increasingly being used in men with clinical suspicion of prostate cancer.
- Recent prospective clinical trials have shown that multiparametric magnetic resonance imaging with intravenous contrast agent as a triage test improved risk stratification of men with clinical suspicion of prostate cancer.
- We have developed a unique rapid biparametric MRI (bpMRI) protocol, called IMPROD bpMRI, that takes only 15 minutes to acquire images and is performed without intravenous contrast agent.
- In our previous single-institution trial (NCT01864135, http://petiv.utu.fi/improd/), IMPROD bpMRI demonstrated high diagnostic performance, thus the current trial aimed to validate IMPROD bpMRI in a large, prospective, multicenter cohort (MULTI-IMPROD trial, NCT02241122) and again provide freely available data to improve MRI quality and standardization (http://petiv.utu.fi/multiimprod/).

**What did the researchers do and find?:**

- Between February 1, 2015, and March 31, 2017, 364 men with a clinical suspicion of prostate cancer were enrolled at 4 institutions in Finland. Men with an equivocal to high suspicion of prostate cancer (IMPROD bpMRI Likert score 3–5) had targeted biopsy followed by systematic biopsy. Men with a low to very low suspicion of prostate cancer (IMPROD bpMRI Likert score 1–2) had only systematic biopsy.
- IMPROD bpMRI demonstrated high negative predictive value for clinically significant prostate cancer (defined as a Gleason score ≥ 3 + 4; Gleason grade group 2 or higher) in both trials (MULTI-IMPROD trial and IMPROD trial).

**What do these findings mean?:**

- The use of IMPROD bpMRI in men with clinical suspicion of prostate cancer limits unnecessary biopsy procedures while maintains or improves detection rate of clinically significant prostate cancer.

## Introduction

Prostate cancer (PCa) is the most common solid organ cancer in men in Europe [1]. In contrast to the detection protocol for many other solid organ cancers, the decision of whom to biopsy among patients with a clinical suspicion of PCa has historically not been based on imaging findings [2]. The standard diagnostic pathway has relied on prostate specific antigen (PSA) levels and digital rectal examination (DRE) findings to determine if a man should undergo transrectal ultrasound-guided (TRUS) biopsies [2]. This pathway is well known to result in unnecessary biopsies, overdetection of nonsignificant PCa (non-SPCa), and underdetection of clinically significant PCa (SPCa) [2].

Two recent prospective trials have shown that an alternative pathway using multiparametric magnetic resonance imaging (MRI) with dynamic contrast enhancement as a triage test reduces unnecessary biopsies, decreases the detection of non-SPCa, and improves the detection of SPCa [2,3]. Based on these trials, some advocate the use of multiparametric MRI with dynamic contrast enhancement as a primary tool in the PCa diagnostic pathway [2,3]. A majority of the published clinical trials and studies [2–7] evaluating pre-biopsy prostate MRI used dynamic-contrast-enhanced MRI (DCE-MRI), except for the recently completed single-institution BIDOC trial [8,9], which was initiated after the start of the MULTI-IMPROD trial (November 2015 versus September 2014). Currently, one of the major challenges limiting wider implementation of multiparametric MRI as a primary detection tool for PCa is insufficient standardization of reporting and imaging techniques. Additionally, the high cost associated with performing an increased number of high-quality prostate MRI studies is a serious barrier for any healthcare system to overcome. Careful development, validation, and ultimately multi-institutional standardization of rapid prostate MRI protocols are urgently needed.

The current trial is an extension to our rigorous research in the MRI physics, imaging repeatability, and clinical applicability of pre-biopsy prostate MRI (e.g., [10–13]). These research efforts have led to the development of a rapid prostate MRI protocol (acquisition time < 15 min) that does not use endorectal coils nor intravenous contrast agents—the IMPROD biparametric MRI (bpMRI) protocol. We emphasize that IMPROD bpMRI is unique and very different from previous prostate MRI studies since diffusion-weighted imaging (DWI) is performed in 3 separate acquisitions. This is in distinct contrast to commonly used single-acquisition methods. IMPROD bpMRI demonstrated a high negative predictive value (NPV) for SPCa (90%, 34/38) in a pre-biopsy setting in the single-institution prospective IMPROD trial [14] (NCT01864135, Improved Prostate Cancer Diagnosis—Combination of Magnetic Resonance Imaging and Biomarkers; http://petiv.utu.fi/improd/).

The aim of the current trial was to validate IMPROD bpMRI in a large, prospective, multicenter cohort (MULTI-IMPROD trial, NCT02241122) and again provide freely available data to improve MRI quality and standardization. Specifically, we aimed to evaluate the accuracy, sensitivity, specificity, NPV, and positive predictive value of IMPROD bpMRI.

## Methods

### Study design and study population

Between February 1, 2015, and March 31, 2017, 364 men with a clinical suspicion of PCa were prospectively enrolled into a prospective, registered validation trial (MULTI-IMPROD, NCT02241122) at 4 different institutions in Finland (in Turku, Pori, Tampere, and Helsinki). Enrolled men were aged 18 years or older, had suspicion of PCa based on 2 repeated PSA measurements ranging from 2.5 to 20.0 μg/l and/or an abnormal DRE. Exclusion criteria were previous prostate biopsy, previous prostate surgery, previous diagnosis of PCa, acute prostatitis, or contraindications for MRI. START consortium reporting standards were followed for the study [15].

### Study end points

The primary outcome of the trial was diagnostic accuracy—including overall accuracy, sensitivity, specificity, NPV, and positive predictive value—of IMPROD bpMRI for SPCa, which was defined as a Gleason score ≥ 3 + 4.

Additional analyses of the trial included the following: (1) NPV for any PCa; (2) the cancer detection rate (CDR) for PCa, SPCa, and non-SPCa using targeted biopsy (TB), systematic biopsy (SB), and their combinations; and (3) detection rates of PCa, SPCa, and non-SPCa in the IMPROD bpMRI Likert score groups.

### MRI protocol and MRI reporting

IMPROD bpMRI was performed using body array coils (no endorectal coil) and 3 Tesla (3 T) MRI scanners in Turku (Verio, Siemens), Tampere (Skyra, Siemens), and Helsinki (Skyra, Siemens). A 1.5 T (Aera, Siemens) MRI scanner was used in Pori. The MRI protocol consisted of T2-weighted acquisitions in axial and sagittal planes, 3 separate DWI sequences, and their corresponding calculated apparent diffusion coefficient maps, fitted using a mono-exponential fit. DCE-MRI was not performed; thus, intravenous contrast agent was not used. DWI datasets were collected in 3 separate acquisitions: (1) *b*-values 0, 100, 200, 300, and 500 s/mm^2^ [16]; (2) *b*-values 0 and 1,500 s/mm^2^; (3) *b*-values 0 and 2,000 s/mm^2^. The imaging protocol was carefully optimized to allow comparable image quality at 1.5 T and 3 T. The overall imaging time using 3 T scanners was 13–17 minutes including shimming and calibration, while the corresponding time at 1.5 T was about 3 minutes longer. Only routinely available MRI acquisition and post-processing methods were used, to allow widespread use of the MRI protocols [17]. The detailed MRI protocols and importable MRI protocols are available at the study server (http://petiv.utu.fi/multiimprod/) as well as at protocols.io (doi: 10.17504/protocols.io.ynifvce). Providing the evidence behind the use of 3 separate DWI acquisitions with the acquisition parameters employed in the IMPROD bpMRI protocol is beyond the scope of the current paper. In short, the 3 separate DWI acquisitions were carefully optimized to maximize the contrast between normal tissues and cancer, and differentiate cancer from susceptibility artifacts, which can decrease the diagnostic performance of prostate DWI performed using echo-planar read-out [17].

All imaging datasets were reported by the local radiologists (AK, OO, and SMV) and confirmed or re-reported centrally by 1 designated central reader (IJ) to guarantee reporting integrity before each biopsy procedure. Following each individual MRI scan, the datasets were uploaded to a central server. At each center, the local radiologist (AK, OO, or SMV) reported each IMPROD bpMRI scan using the same reporting system used in the IMPROD trial [14]. All reports and datasets were uploaded to the central study server within 7 days of the MRI scan. All bpMRI scans were reported centrally, and the local report was approved or modified by 1 designated central reader (IJ, having 6 years of prostate MRI experience at the beginning of the trial, using the IMPROD bpMRI Likert scoring system). If there was disagreement between the central report and the report issued by the local radiologist, the report by the central reader (IJ) was used, to ensure consistency in reporting across all centers. The central reader was unaware of clinical data such as PSA measurements. Prospective MRI reports included sector map drawings in 3 different planes; axial, coronal, and sagittal, containing corresponding key images of the lesion(s). The same IMPROD bpMRI Likert scoring system was used in both the IMPROD (NCT01864135) and MULTI-IMPROD (NCT02241122) trials. The IMPROD bpMRI Likert score used here is a 5-tiered scale describing the likelihood of significant PCa in MRI: (1) SPCa is highly unlikely to be present, (2) SPCa is unlikely to be present, (3) SPCa is possible (equivocal finding), (4) SPCa is likely to be present, or (5) SPCa is highly likely to be present.

### Biopsy procedure

All prostate biopsies were performed transrectally by experienced urologists (*n* = 7) without enema and with periprostatic block. Antibiotic prophylaxis and biopsy-related complications have been recently reported [18,19]. For each man with at least 1 IMPROD Likert score 3–5 lesion, the biopsy procedure started with a TB. Two TB cores were taken from up to 2 lesions. TB was performed with cognitive registration in Turku, Tampere, and Pori and with software registration in Helsinki (UroNav Fusion Biopsy, Invivo Corporation, Brisbane, Australia). TB was followed by a 12-core SB performed by the same operator, who was aware of the MRI results. For men without IMPROD bpMRI Likert score 3–5 lesions, only SB was performed. An additional 2 biopsy cores were taken from all men for the purpose of biomarker research.

### Histopathological analysis

All histopathological biopsies were reported separately (core length, cancer length, Gleason grade) at each center by expert pathologists, each with at least 5 years of experience in genitourinary pathology at the beginning of the trial, using the 2014 International Society of Urological Pathology Modified Gleason Grading System [20]. The overall Gleason score for each patient using TB or SB was assigned as the combination of the most frequent Gleason grade and the highest Gleason grade in the TB and SB cores. MRI findings and clinical data, including PSA values, were not made available to the pathologists.

### Definition of PCa aggressiveness risk groups and SPCa

SPCa was defined as biopsy Gleason score ≥ 3 + 4 (Gleason grade group 2 or higher). In order to facilitate comparison of MULTI-IMPROD trial results with those of prior studies, the analyses in the current trial were also performed using the following 2 additional definitions. Definition 2 [21]: Gleason score of 3 + 4 with ≥50% of any core containing PCa and/or ≥4 SB cores positive for cancer and/or Gleason score of 4 + 3 or higher. Definition 3 [22]: biopsy Gleason score of 4 + 3 or higher. Analyses using these 2 additional definitions are presented on the trial server (http://petiv.utu.fi/multiimprod/) as well as in S1 and S2 Tables.

### Statistical methods

Continuous variables are described as means and standard deviations (SDs) or medians and interquartile ranges (IQRs) as appropriate. The Shapiro–Wilk test was used to check the normality. Pearson’s chi-squared test was used to compare the proportion of men upgraded based on TB compared with SB and vice versa. Categorical variables are presented as frequencies and proportions. Confidence intervals for likelihood ratios are based on the method by Simel et al. [23]. Statistical analyses were conducted using R version 3.2.0 software (R Foundation for Statistical Computing, Vienna, Austria) and JMP and SAS software (SAS, Cary, NC, US).

## Results

**Fig 1:**
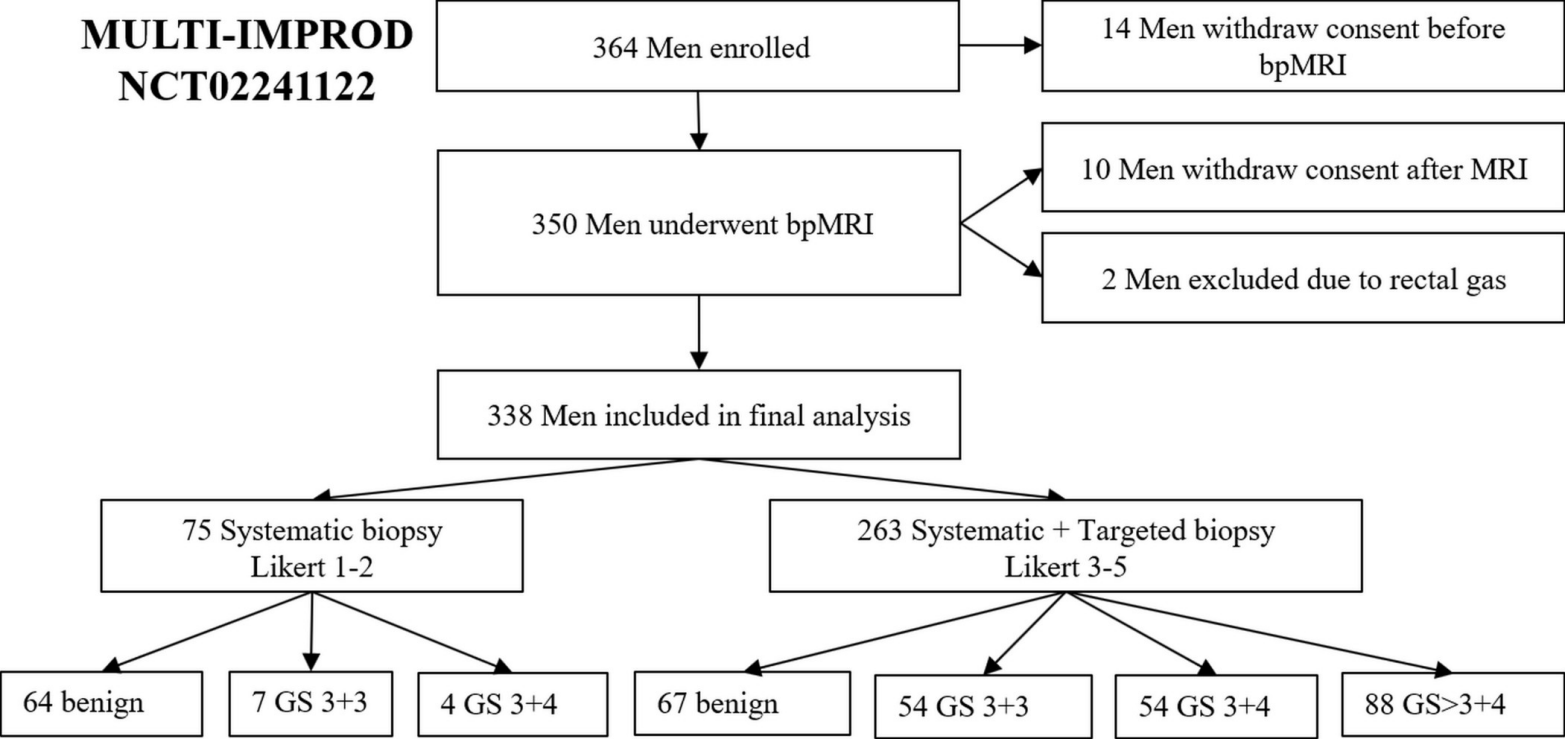
Study flowchart for men undergoing both targeted and systematic biopsy. bpMRI, biparametric magnetic resonance imaging; GS, Gleason score.

Recruitment of the trial population is described in Fig 1. Of the 364 men recruited, 14 withdrew their consent before and 10 after MRI. Two men were excluded due to non-diagnostic IMPROD bpMRI studies caused by rectal-gas-related artifacts on DWI. The majority of the patients who withdrew their consent were either claustrophobic or gave no specific reason for withdrawing.

Patient characteristics of the 338 included men are given in Table 1. The proportion of men included in the final analyses from each center was 38% (130/338) from Turku, 27% (92/338) from Pori, 17% (58/338) from Helsinki, and 17% (58/338) from Tampere. Patient age ranged from 29 to 82 years, with a mean of 64 years. The cohort had a median (IQR) PSA of 6.9 (3.9) μg/l, PSA density of 0.17 (0.12) μg/l/ml, free PSA of 15% (9%), and prostate volume of 39 (25) ml. Thirty-four men (10%, 34/338) had a documented *5*-alpha reductase inhibitor as a concomitant medication, 86 men (25%, 86/338) had suspicious DRE findings, and 85 (25%, 85/338) had suspicious TRUS biopsy findings. Using the IMPROD bpMRI protocol, an overall IMPROD bpMRI Likert score of 1–2 was reported in 75 (22%, 75/338) cases, and IMPROD bpMRI Likert scores of 3–5 were found in 263 (78%, 263/338) patients (Fig 1), of which an IMPROD bpMRI Likert score of 5 was the most frequent finding (41%, 138/338). More than half of the cohort (57%, 192/338) had tumors classified as benign or non-SPCa (Gleason score 3 + 3).

**Table 1:** Patient demographics.

Characteristic	Mean (SD), median (IQR), or *n* (%)
Age, years; mean (SD)	64 (8)
PSA, μg/l; median (IQR)	6.9 (5.1–9.0)
Free PSA, percent; median (IQR)	15 (11–20)
Prostate volume, ml; median (IQR)	39 (30–54)
PSA density, μg/l/ml; median (IQR)	0.17 (0.12–0.24)
5-ARI; *n* (%)	34 (10)
DRE positive; *n* (%)	86 (25)
TRUS biopsy positive; *n* (%)	85 (25)
IMPROD bpMRI Likert score; *n* (%)	
1	34 (10)
2	41 (12)
3	66 (20)
4	59 (17)
5	138 (41)
Biopsy result	
Benign; *n* (%)	131 (39)
Cancer; *n* (%)	
GGG 1/Gleason score 3 + 3	61 (18)
GGG 2/Gleason score 3 + 4	58 (17)
GGG 3/Gleason score 4 + 3	36 (11)
GGG 4/Gleason score 4 + 4/3 + 5/5 + 3	20 (6)
GGG 5/Gleason score 4 + 5/5 + 5	32 (9)

5-ARI, *5*-alpha reductase inhibitor; bpMRI, biparametric magnetic resonance imaging; DRE, digital rectal examination; GGG, 2014 International Society of Urological Pathology Gleason grade group; PSA, prostate specific antigen; TRUS, transrectal ultrasound-guided.

### Diagnostic accuracy

In men with an IMPROD bpMRI Likert score of 1–2, the majority of the biopsies yielded no cancer (85%, 64/75) and only 4 men (5%, 4/75) had SPCa. The prevalence of SPCa increased with increasing IMPROD bpMRI Likert score: SPCa was diagnosed in 5% (2/34), 5% (2/41), 12% (8/66), 39% (23/59), and 88% (121/138) of men with IMPROD bpMRI Likert scores of 1, 2, 3, 4, and 5, respectively (Table 2). The distribution and relationship of IMPROD bpMRI Likert scores and Gleason scores per patient based on TB and SB are shown in Table 2. The NPV for SPCa of IMPROD bpMRI Likert scores 1–2 and 1–3 was 95% (71/75) (95% CI 87%–98%) and 92% (129/141) (95% CI 86%–95%), respectively. Based on these results, 22% (75/338) and 42% (141/338) reductions in the number of men undergoing biopsy would be achieved, and only 4 (3%, 4/146) and 12 (8%, 12/146) men with SPCa would be missed, when restricting biopsy (TB and SB combined) to men with IMPROD bpMRI Likert scores of 3–5 and 4–5, respectively.

**Table 2:** Prevalence of prostate cancer and clinically significant prostate cancer in different IMPROD bpMRI Likert score groups based on a cohort of 338 men.

	IMPROD bpMRI Likert score					Total
	1	2	3	4	5	
**No cancer**	31 (9%)	33 (10%)	42 (12%)	19 (5%)	6 (2%)	131 (38%)
**GGG 1/Gleason score 3 + 3**	1 (0%)	6 (2%)	16 (5%)	17 (5%)	21 (6%)	61 (18%)
**GGG 2/Gleason score 3 + 4**	2 (1%)	2 (1%)	4 (1%)	13 (4%)	37 (11%)	58 (18%)
**GGG > 2/Gleason score > 3 + 4**	0 (0%)	0 (0%)	4 (1%)	10 (3%)	74 (22%)	88 (26%)
**Total**	34 (10%)	41 (12%)	66 (20%)	59 (17%)	138 (41%)	338 (100%)

Clinically significant prostate cancer: Gleason score 3 + 4 or higher.

bpMRI, biparametric magnetic resonance imaging; GGG, 2014 International Society of Urological Pathology Gleason grade group.

The diagnostic performances of IMPROD bpMRI in the current trial (MULTI-IMPROD trial) and in the pre-validation cohort (IMPROD trial) are shown in Table 3. As with the pre-validation cohort (IMPROD trial), the sensitivity and NPV of IMPROD bpMRI (IMPROD bpMRI Likert score 1–2 versus 3–5) for the detection of benign or non-SPCa (Gleason score 3 + 3) versus SPCa (Gleason score 3 + 4 or higher) were high, 97% (142/146) and 95% (71/75), respectively.

**Table 3:** Comparison between the current trial (MULTI-IMPROD trial) and the pre-validation cohort (IMPROD trial).

Measure	MULTI-IMPROD trial	IMPROD trial
Trial duration	February 2015–May 2017	March 2013–February 2015
Trial registration	NCT02241122	NCT01864135
Patient cohort in total	338	161
Participating centers	Turku, Pori, Tampere, Helsinki	Turku
Age, mean (SD), years	64 (8)	65 (8)
PSA, median (IQR), μg/l	6.9 (3.9)	7.5 (3.9)
IMPROD bpMRI Likert score		
1–2	75 (22%, 75/338)	38 (23%, 38/161)
3	66 (20%, 66/338)	24 (14%, 24/161)
4–5	197 (58%, 197/338)	99 (61%, 99/161)
Sensitivity*	97% (142/146) [93%–99%]	95% (79/83) [88%–98%]
Specificity*	37% (71/192) [31%–44%]	44% (34/78) [33%–55%]
NPV*	95% (71/75) [87%–98%]	90% (34/38) [76%–96%]
PPV*	54% (141/263) [48%–60%]	64% (79/123) [55%–72%]
Accuracy*	63% (217/338)	70% (113/161)

*Sensitivity, specificity, NPV, PPV, and accuracy values are based on binary classification (IMPROD bpMRI Likert score 1–2 versus 3–5) for predicting prostate cancer with Gleason score ≥ 3 + 4. 95% confidence intervals given in brackets.

bpMRI, biparametric magnetic resonance imaging; NPV, negative predictive value; PPV, positive predictive value; PSA, prostate specific antigen.

### Comparison of TB with SB

**Fig 2:**
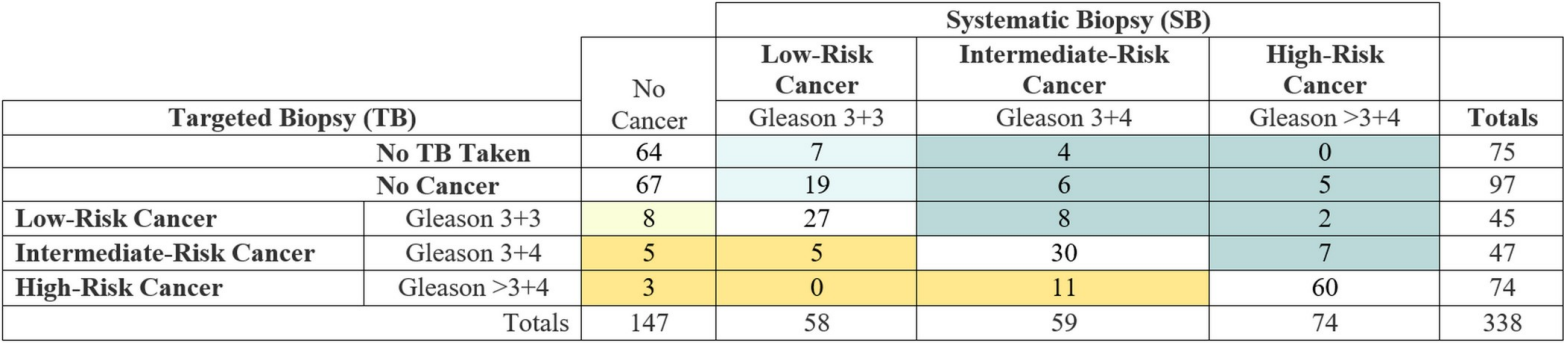
Comparison of biopsy findings from systematic 12-core biopsy and biparametric MRI targeted biopsy (2 cores per lesion, up to 2 lesions per man). Biopsy findings per patient of systematic 12-core biopsy compared with IMPROD biparametric MRI targeted biopsy for the total cohort of 338 men. Yellow shading indicates men in whom targeted biopsy upgraded the PCa risk category in relation to systematic 12-core biopsy. Dark yellow indicates cases in which the upgrade was to an intermediate or high-risk category. Teal shading indicates men in whom systematic 12-core biopsy upgraded PCa risk category in relation to targeted biopsy (or no biopsy based on non-suspicious MRI). Dark teal indicates cases in which the upgrade was to an intermediate or high-risk category.

No statistically significant difference was found between IMPROD-bpMRI-based TB compared with SB in terms of CDR for SPCa (36%, 121/338, versus 39%, 133/338, respectively; *p* > 0.05) and Gleason score 3 + 3 (13%, 45/338, versus 17%, 58/338; *p* > 0.05). The comparison of histopathological outcomes of TB versus SB is shown in Fig 2 for a total cohort of 338 men.

In a sub-cohort of 263 men with IMPROD bpMRI Likert scores of 3–5, the CDR of IMPROD-bpMRI-based TB for SPCa was 46% (121/263) compared with 49% (129/263) for SB (*p* > 0.05). Similarly, no statistically significant difference (*p* > 0.05) was found for the CDR for Gleason score 3 + 3 between of IMPROD bpMRI TB and SB (17%, 45/263, and 19%, 51/263, respectively).

## Discussion

In this multi-institutional trial, the clinical utility of a rapid biparametric pre-biopsy MRI (IMPROD bpMRI) in men with a clinical suspicion of PCa has been prospectively validated. IMPROD bpMRI demonstrated a high NPV for SPCa in both trials (MULTI-IMPROD trial [NCT02241122] and IMPROD [NCT01864135]).

The PROMIS [2], PRECISION [3], and MRI-FIRST [24] trials demonstrated that multiparametric MRI including DCE-MRI, when compared to the standard SB pathway as a triage test, could reduce unnecessary biopsies and overdetection of non-SPCa while increasing the CDR for SPCa [25]. However, widespread adoption of the multiparametric MRI triage pathway has many implementation challenges, including long acquisition times and prohibitive costs. The associated cost and time barriers to ensuring MRI availability to all men with a clinical suspicion of PCa are major issues of concern. The recent single-institution BICOD trial [8,9] demonstrated the feasibility of simple bpMRI (axial T2-weighted imaging and single DWI acquisition) in men with a clinical suspicion of PCa in a clinical setting similar to that of the IMPROD and MULTI-IMPROD trials. BpMRI without DCE-MRI has lower acquisition time (15 versus up to 25–30 minutes), has lower cost, and is faster to perform due to the time saved in patient preparation such as acquiring intravenous access [8,9,26,27]. Adopting bpMRI with faster acquisition times would thus be more feasible on a larger scale. Further cost reduction can be achieved by careful optimization of the IMPROD bpMRI reporting setup, enabling an average reporting time per IMPROD bpMRI study of under 10 minutes, including creation of a report with representative images and a simple scheme with lesion location.

Adopting the pre-biopsy MRI pathway would result in a significant increase in the number of imaging studies performed. To maintain high diagnostic performance, the MRI acquisition protocols and reporting should be standardized [28]. In addition to standardized methodology, proper training of prostate MRI readers and a continuous quality control system are essential. The need for radiologist training is evident as both PROMIS [2] and PRECISION [3] demonstrated only moderate inter-reader agreement [2,3]. In an effort to standardize MRI methodology, we provide free public data access to the IMPROD bpMRI protocol and to all IMPROD and MULTI-IMPROD datasets, MRI reports, and clinical datasets with pathology and follow-up data on our server (IMPROD: http://petiv.utu.fi/improd/; MULTI-IMPROD: http://petiv.utu.fi/multiimprod/). The main goal of the IMPROD and MULTI-IMPROD servers is to function as a teaching and standardization site. Using the imaging data and reports stored in the server, with corresponding histopathological verification (including whole mount prostatectomy sections), prostate MRI readers could improve their skills by practicing prostate MRI reporting using the IMPROD bpMRI protocol. The most important benefit of the server is free access to an optimized MRI protocol that can be used at clinical 1.5 T and 3 T MRI scanners, providing high-quality prostate MRI for men with elevated PSA.

The IMPROD bpMRI protocol development, which put strong emphasis on MRI physics (e.g., [10–13]), was independent of and parallel to the development of the PI-RADS guidelines of the European Society of Urogenital Radiology. In fact, during 2010–2014, the IMPROD bpMRI protocol development was in sharp contrast to the PI-RADS version 1 guidelines in that the IMPROD bpMRI protocol stressed the added value of DWI performed using optimized high *b-*values in addition to low *b*-values for monoexponential DWI quantification. Interestingly, PI-RADS version 2 from 2014 and version 2.1 from 2019 [29], with their emphasis on DWI data collected using high *b*-values, are similar to the IMPROD bpMRI reporting system, which was developed in 2012–2013, before the start of the IMPROD trial (NCT01864135). IMPROD bpMRI put emphasis on the careful optimization of prostate MRI acquisition and post-processing parameters. Between 2010 and 2013, we explored the added value of DCE-MRI and proton magnetic resonance spectroscopy (^1^H-MRS) performed using Point RESolved Spectroscopy (PRESS) sequence in men with a clinical suspicion of PCa. Both DCE-MRI and PRESS-based ^1^H-MRS demonstrated limited added value to optimized DWI [16]. However, these findings were explored in a small 2-institution study involving only 55 men [16], and no comparative arm using these methodologies was included in our current trial.

The main limitation of both the IMPROD and MULTI-IMPROD trials is uncertainty over the true PCa and SPCa prevalence in the study cohorts, since the men did not undergo saturation biopsy and only part of each cohort underwent radical prostatectomy. In addition, men had different biopsy protocols (SB versus SB + TB) based on MRI risk estimation. Therefore, this introduces a potential bias (partial verification bias) since men with high MRI risk score had more extensive biopsies [30]. The bias would be relevant if men with low MRI risk score (IMPROD bpMRI Likert score 1–2) had a significant quantity of undetected cancers. However, we feel that this is unlikely based on the following rationale. First, IMPROD bpMRI enabled the detection of 93% (37/40) of lesions with Gleason score > 3 + 4 in men enrolled in the pre-validation IMPROD trial who underwent prostatectomy [31]. Second, all men enrolled as part of the IMPROD and MULTI-IMPROD trials are underdoing close follow-up. In the IMPROD trial cohort, the current median follow-up time is 37 months. In 49 men with IMPROD bpMRI Likert scores of 1–3, only 2 men (4%, 2/49) were found to have low-volume PCa with a Gleason score of 3 + 4 during the follow-up period, suggesting that the initial IMPROD bpMRI scan had a high NPV.

Although MRI reports were centrally approved or reported, inter-reader variability was not assessed. Central reporting may also limit generalizability, but the study server, providing access to all imaging datasets and MRI reports, is a teaching tool for other readers reporting prostate MRI. All prospectively reported MRI examinations are freely available on the study server, allowing further retrospective analyses by interested parties. Histopathology material was reported locally without central reviewing, strictly adhering to international standards.

Transrectal TB and SB were performed during single sessions by the same urologist, possibly affecting the trajectories of SB and increasing CDR for SB. However, all of the urologists performing TB and SB strictly followed the SB sampling scheme to limit the possible introduction of bias. Transperineal biopsies are increasing in popularity [25], partly because of an increased CDR and a decreased rate of infection. In MULTI-IMPROD, all centers performed transrectal biopsies exclusively.

Finally, both MULTI-IMPROD (NCT02241122) and IMPROD (NCT01864135) are limited in terms of a lack of ethnic diversity in the patient population due to the geographical nature of this study.

### Conclusion

In this prospective multicenter trial, a previously developed unique MRI acquisition and reporting protocol, IMPROD bpMRI (NCT01864135), enabled the detection of SPCa in 97% (142/146) of men with a Gleason score ≥ 3 + 4 and demonstrated a NPV of 95% (71/75; 95% CI 87%–98%). IMPROD bpMRI consists of T2-weighted imaging and 3 separate DWI acquisitions with an acquisition time less than 15 minutes. Wider implementation of the IMPROD bpMRI protocol with TB could enable improved PCa risk stratification by reducing the number of prostate biopsies while simultaneously increasing the detection of SPCa. Moreover, IMPROD and MULTI-IMPROD are the first prospective imaging clinical trials to our knowledge providing free public access to all imaging datasets and to clinical and histopathology findings, enabling the external validation and implementation of IMPROD bpMRI by other centers. IMPROD bpMRI has demonstrated its proficiency as a powerful and efficient tool for improved PCa risk stratification in men with a clinical suspicion of PCa based on elevated PSA and/or DRE.

## Supporting information

S1 CONSORT Checklist (DOC)

S1 Table Comparison between the current trial (MULTI-IMPROD trial) and the pre-validation cohort (IMPROD trial) using definition 2 of clinically significant prostate cancer: Gleason score of 3 + 4 with ≥50% of any core containing prostate cancer and/or ≥4 SB cores positive for cancer and/or Gleason score of 4 + 3 or higher. (DOCX)

S2 Table Comparison between the current trial (MULTI-IMPROD trial) and the pre-validation cohort (IMPROD trial) using definition 3 of clinically significant prostate cancer: biopsy Gleason score of 4 + 3 or higher. (DOCX)

## Abbreviations

bpMRI: biparametric magnetic resonance imaging
CDR: cancer detection rate
DCE-MRI: dynamic-contrast-enhanced magnetic resonance imaging
DRE: digital rectal examination
DWI: diffusion-weighted imaging
MRI: magnetic resonance imaging
non-SPCa: nonsignificant prostate cancer
NPV: negative predictive value
PCa: prostate cancer
PSA: prostate specific antigen
SB: systematic biopsy
SPCa: significant prostate cancer
TB: targeted biopsy
TRUS: transrectal ultrasound-guided
