# Supplementary material for: Validation of IMPROD biparametric MRI in men with clinically suspected prostate cancer: A prospective multi-institutional trial
Source: PLoS Med. 2019 Jun 3;16(6):e1002813. doi: 10.1371/journal.pmed.1002813 (PMC6546206; doi:10.1371/journal.pmed.1002813)
Supplement: S1 Table — (DOCX) [file pmed.1002813.s002.docx]

**S1 Table** Comparison between the current trial (MULTI-IMPROD trial) and the pre-validation cohort (IMPROD trial) using the Definition no.2 of clinically significant prostate cancer: Gleason score of 3+4 with ≥ 50% of any core containing and/or ≥ 4 SB cores positive for cancer and/or Gleason score of 4+3 or higher.

|  | **MULTI-IMPROD trial** | **IMPROD trial** |
| --- | --- | --- |
| **Sensitivity*** | 99% (136/137) [96-100%]^#^ | 97% (70/72) [90-99%]^#^ |
| **Specificity*** | 37% (74/201) [31-44%]^#^ | 40% (36/89) [31-51%]^#^ |
| **NPV*** | 99% (74/75) [93-100%]^#^ | 95% (36/38) [83-99%]^#^ |
| **PPV*** | 52% (136/263) [46-58%]^#^ | 57% (70/123) [48-65%]^#^ |
| **Accuracy*** | 62% (210/338) | 66% (106/161) |
