# Supplementary material for: Validation of IMPROD biparametric MRI in men with clinically suspected prostate cancer: A prospective multi-institutional trial
Source: PLoS Med. 2019 Jun 3;16(6):e1002813. doi: 10.1371/journal.pmed.1002813 (PMC6546206; doi:10.1371/journal.pmed.1002813)
Supplement: S2 Table — (DOCX) [file pmed.1002813.s003.docx]

**S2 Table** Comparison between the current trial (MULTI-IMPROD trial) and the pre-validation cohort (IMPROD trial) using the Definition no.3 of clinically significant prostate cancer: Biopsy Gleason score of 4+3 higher.

|  | **MULTI-IMPROD trial** | **IMPROD trial** |
| --- | --- | --- |
| **Sensitivity*** | 100% (88/88) [96-100%]^#^ | 100% (38/38) [91-100%]^#^ |
| **Specificity*** | 30% (75/250) [25-36%]^#^ | 31% (38/123) [23-40%]^#^ |
| **NPV*** | 100% (75/75) [95-100%]^#^ | 100% (38/38) [91-100%]^#^ |
| **PPV*** | 34% (88/263) [28-39%]^#^ | 31% (38/123) [23-40%]^#^ |
| **Accuracy*** | 48% (163/338) | 47% (76/161) |
